# Present-day and future projection of East Asian summer monsoon in Coupled Model Intercomparison Project 6 simulations

**DOI:** 10.1371/journal.pone.0269267

**Published:** 2022-06-03

**Authors:** Min-Ah Sun, Hyun Min Sung, Jisun Kim, Jae-Hee Lee, Sungbo Shim, Young-Hwa Byun

**Affiliations:** Climate Change Research Team, National Institute of Meteorological Sciences, Jeju, Republic of Korea; Institute of Oceanology Chinese Academy of Sciences, CHINA

## Abstract

The East Asian summer monsoon (EASM) is an influential monsoon system that provides two-thirds of the annual precipitation in the Asian region. Therefore, considerable attention has been paid to the changes in future climate. Thus far, studies on EASM characteristics have not been conducted considering specific global warming level (GWL) using Coupled Model Inter-comparison Project 6 (CMIP6) simulations. We analyze the EASM characteristics in present-day (PD) and the changes in EASM corresponding to the projections at 1.5, 2.0, and 3.0°C GWLs. The newly released 30 CMIP6 models effectively captured the migration of the monsoon in PD with a pattern correlation coefficient of 0.91, which is an improvement over that reported in previous studies. As a result of the separate analysis of the P1 (first primary peak; 33–41 pentad) and P2 (from P1 to the withdrawal; 42–50 pentad) periods, a higher frequency of weak to moderate precipitation in P2 and a smaller amount of moderate to extreme precipitation in P1 are mainly occurred. The CMIP6 models project increasing precipitation of approximately 5.7%°C^−1^, 4.0%°C^−1^, and 3.9%°C^−1^ for the three GWLs, respectively, with longer durations (earlier onset and delayed termination). Under the three GWLs, the projected precipitation frequency decreases below 6 mm d^−1^ (76th percentile) and significant increases above 29 mm d^−1^ (97th percentile). These changes in precipitation frequency are associated with an increasing distribution of precipitation amount above 97th percentile. Additionally, these tendencies in P1 and P2 are similar to that of the total period, while the maximum changes occur in 3.0°C GWL. In particular, future changes in EASM accelerate with continuous warming and are mainly affected by enhanced extreme precipitation (above 97th percentile). Our findings are expected to provide information for the implementation of sustainable water management programs as a part of national climate policy.

## Introduction

The East Asian summer monsoon (EASM) precipitation is responsible for two-thirds of the annual precipitation of East Asia (EA) and provides water resources for agriculture and human society [1–6]. Also, EASM precipitation leads water-related disaster due to large variation and extreme events [7, 8]. Thus, projecting and understanding how the EASM precipitation may change in the future climate are fundamentally important issue in sustainable water resource management and infrastructure planning of disaster mitigation.

Reliable projection is also important in research community. Climate projections are generally based on scenarios and climate model is effective tool to project future climate. The Coupled Model Intercomparison Project phase 6 (CMIP6) has designed with new shared socioeconomic pathway (SSP) scenarios [8, 9]. This SSP scenarios in CMIP6 have a clear description of the mitigation and adaptation options in future society. Considering national climate policies need future projection of EASM using new scenarios, the analysis results of CMIP6 models provide more reliable projection than previous CMIP [10–13]. Many studies have reported the future projection of EASM in warmer climate [1, 5, 6, 14–17] and these studies have generally concluded that global warming lead increased precipitation. This increase is mainly attributed to enhancement of thermodynamic effect [18–22] and the role of western North Pacific subtropical high [3, 7, 16, 23, 24]. One of the major phenomena linked with future changes of EASM is the enhanced extreme precipitation [3, 25]. Therefore, representing characteristics of precipitation extremes in warmer climate has been focused on climate change research. However, this study aims to provide information of the changes in the distribution (frequency and amount) of precipitation in EASM period, while previous studies mainly reported enhanced extreme precipitation using related extreme indices. Additionally, sub-seasonal changes in the distribution of precipitation in EASM period are investigated in this study. This information is major issue in sustainable water resource management (e.g. agriculture policy, water-related disaster prevention).

Based on the assessment of multiple evidences, global warming of 2.0°C relative to pre-industrial level would be exceeded during the 21st century. Considering this, future projection under specific global warming level (GWL) are based on novel emergent constraint correction methods [8]. Despite recent projection of EASM, future changes under different warming targets using updated CMIP6 models are still needed further investigation. The main objective of this study is to provide the characteristics of changes in EASM at 1.5°C, 2°C, and 3°C GWLs using newly available CMIP6 model. The analysis results in this study are expected to inform policymaker’s understanding, and to support formation of mitigation and adaptation strategies for summer precipitation changes in East Asia region. Paper is organized as follows. Section 2 describes reanalysis data, CMIP6 datasets, and analysis methods. Results are provided in Section 3 for the simulation of the EASM and its projected changes for a warming of 1.5, 2.0, and 3.0°C. The discussion and conclusions are presented in Sections 4.

## Data and methodology

### Data

Daily precipitation data are obtained from the ERA5 reanalysis data [26], which is the latest generation reanalysis of the European Centre for Medium-Range Weather Forecasts. The resolution of the original ERA5 dataset is 0.25° for both latitude and longitude. To facilitate a comparison between each CMIP6 model simulation and reanalysis, datasets are interpolated in a 1.875° × 1.25° (latitude × longitude) horizontal grid using conservative interpolation.

Daily precipitation data are used for the first realization (only one member) from 30 CMIP6 model ensembles (Table 1) that are available at the time of initializing this study. This process can reduce potential systematic bias in the calculation of MME [27]. For each CMIP6 model, historical simulations and future projections under the SSP-based scenarios from Scenario Model Inter-comparison Project (ScenarioMIP) [9] are used in this study. For future projections, four SSP scenarios: SSP1-2.6, SSP2-4.5, SSP3-7.0, and SSP5-8.5, according to the Tier 1 protocol are used. Historical simulations for the reference period of 1995–2014 are used in this study to determine the present-day (PD) period. All the model datasets are re-gridded to 1.875° × 1.25° (latitude × longitude) before the MME by using conservative interpolation in precipitation. We use the target region for analysis over the EA domain (20–50°N, 110–140°E).

**Table 1:** Description of the Coupled Model Intercomparison Project Phase 6 (CMIP6) climate models used in this study.

Climate Model	Institution	Resolution (latitude × longitude)
ACCESS-CM2	The Commonwealth Scientific and Industrial Research Organization (CSIRO), Australia	144 × 192
ACCESS-ESM1-5		145 × 192
BCC-CSM2-MR	Beijing Climate Center (BCC), China	160 × 320
CAMS-CSM1-0	Chinese Academy of Meteorological Sciences (CAMS), China	160 × 320
CESM2	National Center for Atmospheric Research (NCAR), USA (	192 × 288
CESM2-WACCM		192 × 288
CMCC-CM2-SR5	Fondazione Centro Euro-Mediterraneo sui Cambiamenti Climatici (CMCC), Italy	192 × 288
CNRM-CM6-1	Centre National de Recherches Meteorologiques/ Centre European de Recherche et Formation Avancees en Calcul Scientifique (CNRM/CERFACS), France	128 × 256
CNRM-CM6-1-HR		360 × 720
CNRM-ESM2-1	Centre National de Recherches Meteorologiques/Centre European de Recherche et Formation Avancees en Calcul Scientifique (CNRM/CERFACS), France	128 × 256
CanESM5	Canadian Centre for Climate Modeling and Analysis (CCCma), Canada	64 × 128
EC-Earth3	European Consortium of various institutions (EC), EU	256 × 512
EC-Earth3-Veg		256 × 512
FGOALS-g3	Chinese Academy of Sciences (CAS), China	80 × 180
GFDL-ESM4	National Oceanic and Atmospheric Administration Geophysical Fluid Dynamics Laboratory (NOAA GFDL), USA	180 × 288
HadGEM3-GC31-LL	Met Office Hadley Centre (MOHC), UK	144 × 192
IITM-ESM	Centre for Climate Change Research, Indian Institute of Tropical Meteorology Pune (CCCR-IITM), India	94 × 192
INM-CM4-8	Institute of Numerical Mathematics of the Russian Academy of Sciences (INM), Russia	120 × 180
INM-CM5-0		120 × 180
IPSL-CM6A-LR	Institute Pierre-Simon Laplace (IPSL), France	143 × 144
KACE-1.0-G	National Institute of Meteorological Sciences/Korea Meteorological Administration (NIMS-KMA), Korea	144 × 192
MIROC-ES2L	Atmosphere and Ocean Research Institute (University of Tokyo), Japan	64 × 128
MIROC6	National Institute for Environmental Studies, and Japan Agency for Marine-Earth Science and Technology (MIROC), Japan	128 × 256
MPI-ESM1-2-HR	Max Plank Institute for Meteorology (MPI-M), Germany	192 × 384
MPI-ESM1-2-LR		96 × 192
MRI-ESM2-0	Meteorological Research Institute (MRI), Japan	160 × 320
NESM3	Nanjing University of Information Science and Technology (NUIST), China	96 × 192
NorESM2-LM	Bjerknes Centre for Climate Research (BCCR), Norway	96× 144
NorESM2-MM		192 × 288
UKESM1-0-LL	Natural Environment Research Council and the Met Office Hadley Centre (MOHC), UK	144 × 192

### Characteristics of East Asian summer monsoon

For the analysis of EASM characteristics (onset, withdrawal, and duration), we used the method of Wang and LinHo [28], which calculates the monsoon season using pentad mean precipitation. The pentad mean precipitation is first smoothed to a running mean of five days, and monthly mean precipitation in January is subtracted from each pentad. Then, the first pentad exceeding 5 mm d^−1^ in this series is defined as the onset, and a pentad of less than 5 mm d^−1^ after the onset is defined as the withdrawal. The difference between the withdrawal and onset days is defined as the monsoon duration. Using the threshold method, which defines the onset and withdrawal of the monsoon, is reasonable because precipitation varies according to the regional location [4, 29].

**Fig 1:**
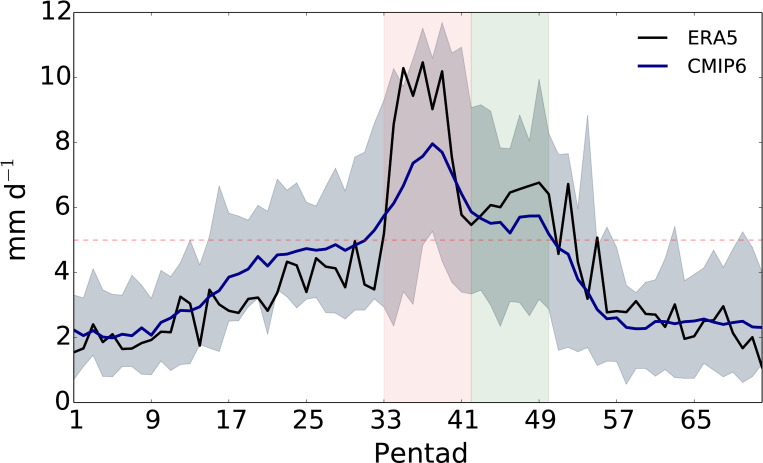
Time series of the pentad mean precipitation anomalies (mm d^−1^) relative to climatology of January from ERA5 (black) and CMIP6 (blue) ensembles. The grey shading indicates the ensemble spread of CMIP6 models. The red and green vertical shadings indicate the P1 and P2 period, respectively.

Many previous studies have shown that the EASM features the first peak (active phase), break, and second peak (revival phase) [30, 31]. The spatiotemporal variability associated with the EASM pattern makes trend analysis difficult and produces geographically inconsistent results [32, 33]. Considering this, we have classified the EASM into two periods (Fig 1), and this approach could consider the sub-seasonal structures [30]. The period with the first primary peak is defined as P1 (33–41 pentad), and from the end of P1 to the withdrawal pentad is defined as P2 (42–50 pentad). This is comparable to the previously defined monsoon season [30, 31, 34–36].

Additionally, changes in daily precipitation frequency and amount distributions of the EASM are analyzed. These distributions reveal the precipitation rates that occur most frequently and contribute the most to the total accumulated precipitation. In this study, a logarithmic bin spacing is applied following Pendergrass and Hartmann [37] to investigate the full range of rates across orders of magnitude from drizzle to extreme. We have used the starting dry threshold of 0.1 mm d^−1^ and there are approximately 100 bins with span rates from 0.1 to 1000 mm d^−1^.

### Specific warming thresholds

As in previous IPCC reports, the pre-industrial period generally referred to as the duration between 1850 and 1900 [38] and it has been widely employed in climate change studies [12, 39–42] and recently recommended by IPCC’s Sixth Assessment Report [8]. To define the time of 1.5, 2.0, and 3.0°C GWLs above pre-industrial level in the individual CMIP6 model, a four-step strategy is adopted to make the threshold crossing estimates comparable across scenarios. First, the time series of the global mean surface temperature is smoothed by a 21-year moving average in all SSP scenarios, and the threshold is considered the centered 21-year period. Second, only the scenarios that have more than 80% of related CMIP6 models are selected to make MME at three GWLs because several scenarios do not reach a higher GWL in the 21^st^ century. For example, the ensemble of 3°C GWL does not have the SSP1-2.6 scenario because there is no model to reach 3°C threshold in it [42]. This approach could make a better ensemble of specific GWLs and reduce uncertainty when comparing the various scenarios considered. Third, the corresponding climate models with selected scenarios are filtered with 5–95% confidence ranges. Finally, to determine a relatively stable climate condition, both the 9-year period (before the selected year in the first step) and the 10-year period (after the selected year in the first step) are compared with the PD period to assess the changes in climate change. Therefore, the ensemble numbers are 90, 66, and 43, respectively, for the 1.5, 2.0, and 3.0°C GWLs under the four SSP scenarios, respectively. Also, period ranges corresponding to the 1.5, 2.0, and 3.0°C GWLs are 2013–2051, 2027–2073, and 2047–2085, respectively. This approach incorporates many model results as possible and reduces uncertainty in model response, which increases in the scenarios corresponding to higher GWL over time in the 21^st^ century [42].

## Results

### Present-day climatology of East Asian summer monsoon

The EASM is characterized by seasonal transition of winds, and the rainband propagates northward from South Asia to the subtropical western North Pacific during summer [43–48]. It is important to simulate these features well within climate models to improve the understanding of EASM and to reduce the uncertainty of future hydrological circulation in the Asian region. Therefore, we first analyze the observed and simulated features of the EASM in this section.

**Fig 2:**
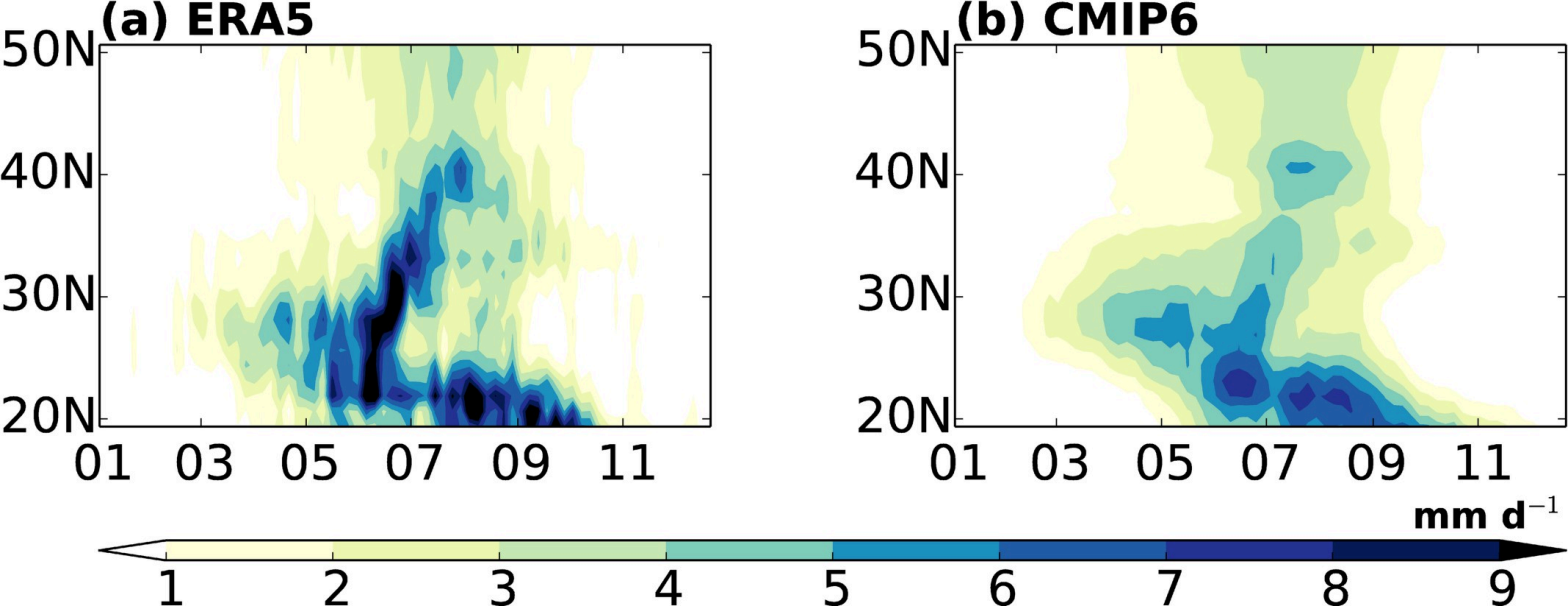
Time–latitude cross sections of pentad mean precipitation anomalies (mm d^−1^) relative to climatology of January, which is averaged over EA domain (20–50°N, 110–140°E) of (a) ERA5 and (b) CMIP6 ensembles for the period 1995–2014 (PD period). The x-axis label indicates the beginning of the month.

Fig 2 shows the time–latitude cross section of pentad averaged precipitation anomalies relative to climatology of January from the ERA5 and CMIP6 ensemble mean, which represents the seasonal migration of the EASM. A significant rainband appears in spring (Fig 2A), although the precipitation amount is relatively lower than that in summer [49–51]. Rainband expansion is associated with increased moisture and the northward advance of the Pacific anticyclone. Subsequently, a significant northward movement is observed around mid-June, where the rainband abruptly progressed northward to northern China and the Korean Peninsula [1, 52, 53]. The EASM begins to retreat after mid-August as the rainband moves southward with a relatively weaker precipitation. Overall, the composite map of the CMIP6 ensemble captures the observed northward migration of the rainband, including the spring rainband in southern China and the following active phase, with a pattern correlation coefficient of 0.91 (Fig 2B). The simulated rainband in spring is slightly stronger than that observed. Similar to previous reports, the rainband over 30°N–40°N is significantly weaker than that in the observation as CMIP6 models generally show weakly simulated northward movement patterns (not shown) [43, 54–56]. The simulated rainband in spring is slightly stronger than that observed. Similar to previous reports, the rainband over 30°N–40°N is significantly weaker than that in the observation as CMIP6 models generally show weakly simulated northward movement patterns (not shown) [43, 54–56]. Accordingly, the simulated onset and withdrawal over the Asian domain tend to be slightly earlier than the observed, but the duration is similar (summarized in Table 2). The total and maximum amounts of precipitation during the EASM period are weaker than those observed (Table 2).

**Table 2:** The values for the characteristics of EASM.

	Onset (pentad)	Withdrawal (pentad)	Duration (pentad)	Amount (mm d^−1^)	Maximum (mm d^−1^)
ERA5	34.5	45.6	11.1	351.8	39.8
CMIP6	34	45.2	11.2	312.5	36.5

**Fig 3:**
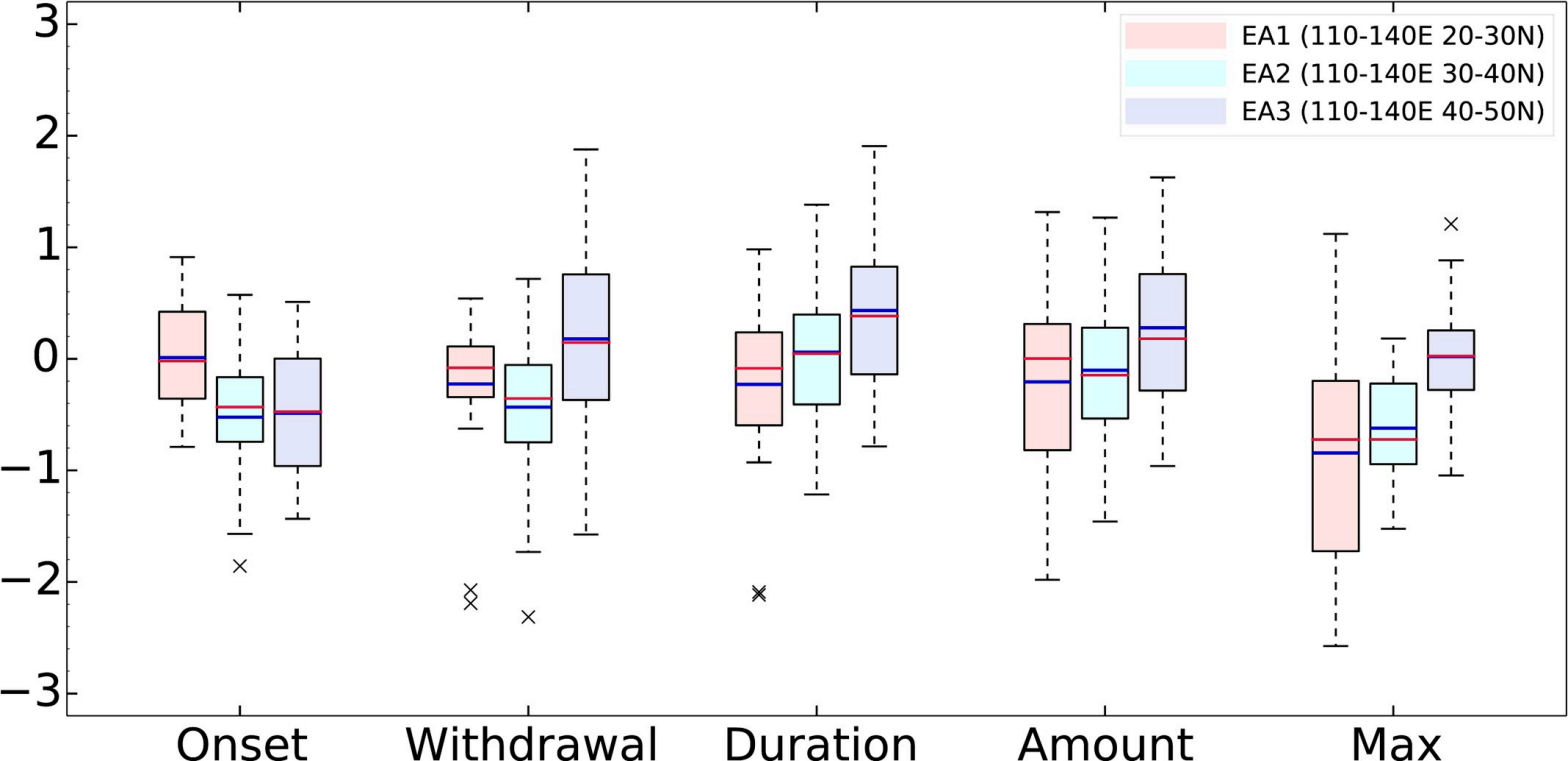
Normalized 30 CMIP6 model biases relative to observations for the EASM characteristics in Table 1. Analysis sub-regions are EA1 (20–30°N), EA2 (30–40°N), and EA3 (40–50°N). Biases are normalized by the observation of each variable. Blue and red bars indicate mean and median values, respectively.

To analyze the detailed local evolution of the monsoon system, we have composed three sub-regions (20°N–30°N, 30°N–40°N, and 40°N–50°N) and investigated the evolution of the EASM. As shown in Fig 3, the model bias is normalized by the observed value, and a negative value indicates that the simulated value is lower than the observed value. To the south of 30°N, the simulated delayed onset and earlier withdrawal have a shorter duration than that observed. In the 30°N–40°N region, early onset and termination with significantly large amplitude of bias in the model have little impact (similar to that observed) on the simulated duration (Fig 3). The longer duration north of 40°N is due to both early onset and late termination. Considering the larger amplitude of amount biases, smaller precipitation amount to the south of 40°N is related to less rain over the East China Sea toward the Korean Peninsula, with the simulated rainband location shifting to the north (Fig 2B). In addition, the simulated maximum precipitation is underestimated compared to that observed south of 40°N, which is similar to previous studies. These results indicate that CMIP6 models still have uncertainty with respect to the EASM rainband and its movement; however, in general, the performance with a good correlation of 0.91 is an improvement over the results of previous CMIP studies [57 (0.79), 13 (0.88)].

**Fig 4:**
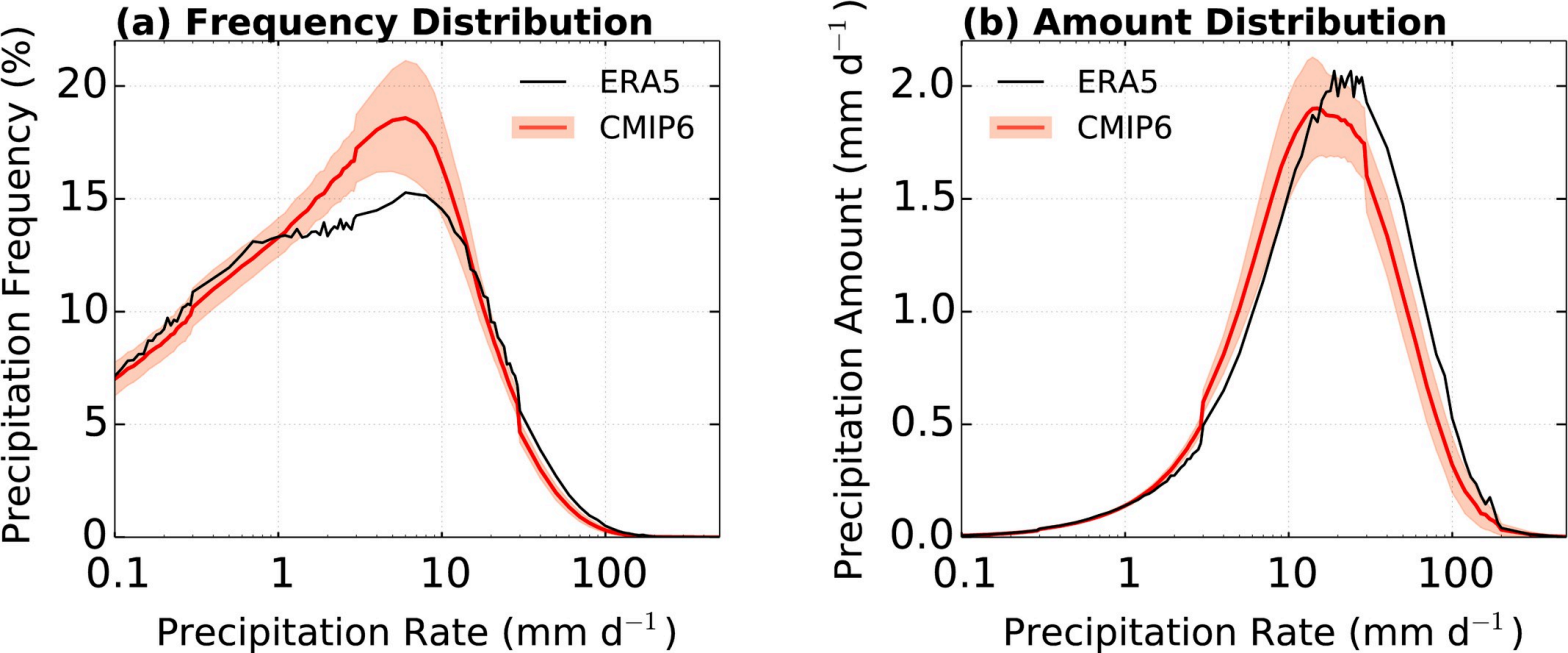
Daily precipitation over the EA domain for distributions of (a) precipitation frequency (%) and (b) precipitation amount (mm d^−1^) in ERA5 and CMIP6 ensembles. The red shading is 95% confidence in the CMIP6 ensemble. Black and red lines indicate ERA5 and CMIP6 ensemble, respectively.

**Fig 5:**
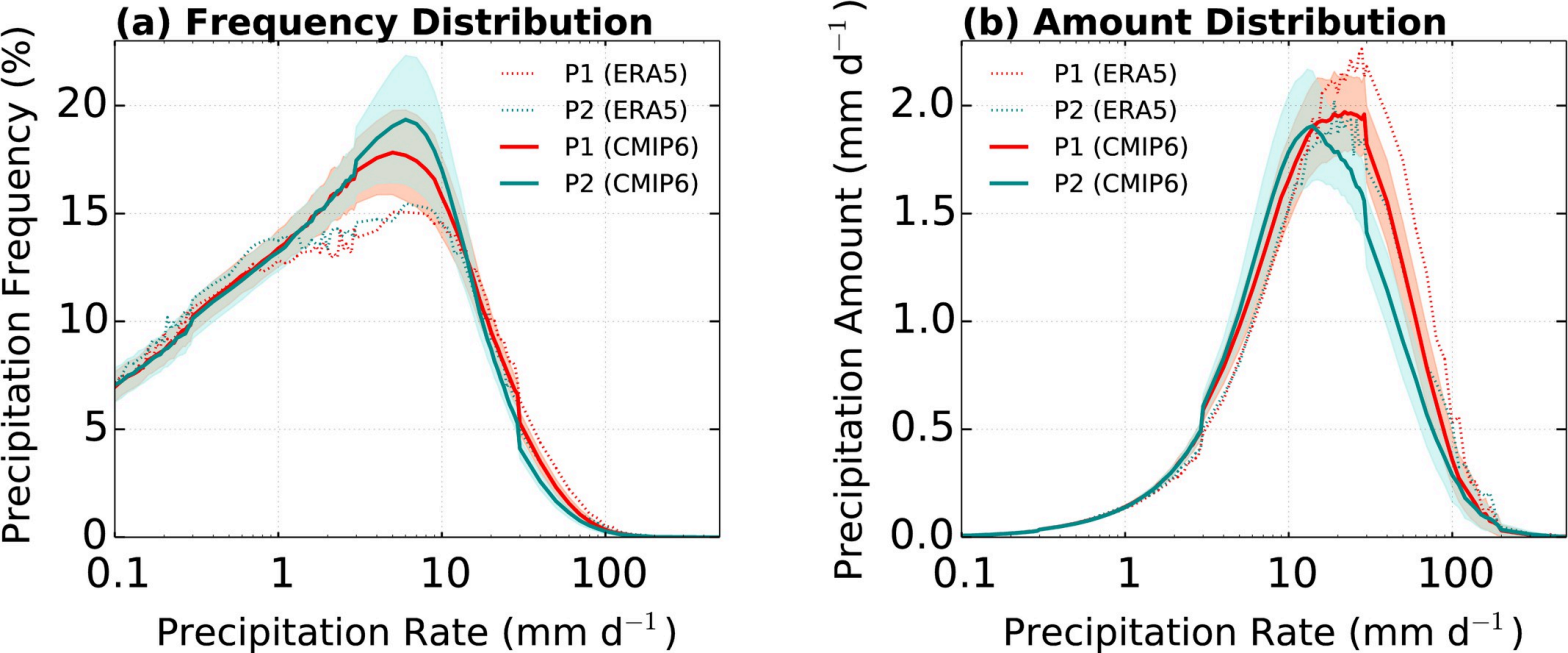
Daily precipitation over the EA domain for distributions of (a) precipitation frequency (%) and (b) precipitation amount (mm d^−1^) in ERA5 and CMIP6 ensembles. The shading is 95% confidence in the CMIP6 ensemble. Red and blue lines indicate P1 and P2, respectively. Solid and dashed lines indicate CMIP6 and ERA5 ensembles, respectively.

Fig 4 shows the comparison between distributions of the daily precipitation frequency and amount over the EA domain (with percentile) from ERA5 and CMIP6 ensemble. The advantage of this approach is that it reduces the effects of bias in precipitation amounts between the different climate models while retaining reliable information about precipitation patterns and behaviors [58]. In Fig 4A, the distributions of precipitation frequency of ERA5 and CMIP6 ensemble agree as both peak at approximately 6 mm d^−1^ (approximately 75th percentile) with a significantly higher simulated frequency in the 1–16 mm d^−1^ (40–92th percentile) range, while the simulated distribution of precipitation frequency below 1 mm d^−1^ and above 16 mm d^−1^ are lower than the observed. This bias at above 16 mm d^−1^ (92th percentile) is associated with a significantly underestimated distribution of precipitation amount (Fig 4B). Fig 5 shows the distributions of precipitation frequency and amount as a function of the precipitation rate for P1 and P2. In Fig 5A, the distributions of frequencies of the ERA5 and CMIP6 ensemble for both periods agree with the peak approximately at 6 mm d^−1^ (approximately 75th percentile), which is similar to that of the total period (Fig 4A). In addition, the simulated precipitation frequency of 1 to 16 mm d^−1^ (40–92th percentile) in both periods are higher than the observed. The climate models generate precipitation through convection and large-scale physics processes. In particular, light precipitation is generated by a convection scheme. Therefore, this analysis means that light precipitation biases produced by a convection scheme [59, 60]. These analysis results indicate that the distributions of precipitation frequency highlight the persistent issue wherein the CMIP6 models simulate more frequently below a moderate precipitation rate [36, 61]. The weak precipitation frequency (below 1 mm d^−1^; 40th percentile) in P2 is significantly higher than that in P1. However, above 16 mm d^−1^, the simulated frequency in P1 is higher than that in P2. In particular, the simulated tendency in P2 has a larger influence on the EASM characteristics in total period than that in P1. In Fig 5B, the CMIP6 ensemble exhibits a larger (significantly smaller) precipitation amount than ERA5 below (above) a moderate rate (approximately 16 mm d^−1^). The simulated peak of the precipitation amount distribution is smaller than that of ERA5 because of the lower distribution of precipitation frequency, which is similar to that of the total period (Fig 4B). These biases in P1 are larger than that in P2. These results are evident in the considerably dry bias of the total amount of domain-averaged precipitation (Table 2) because precipitation from moderate to extreme rate produces the most accumulated precipitation amount [62].

### Projected changes in the EASM under 1.5, 2.0, and 3.0°C of warmings

**Fig 6:**
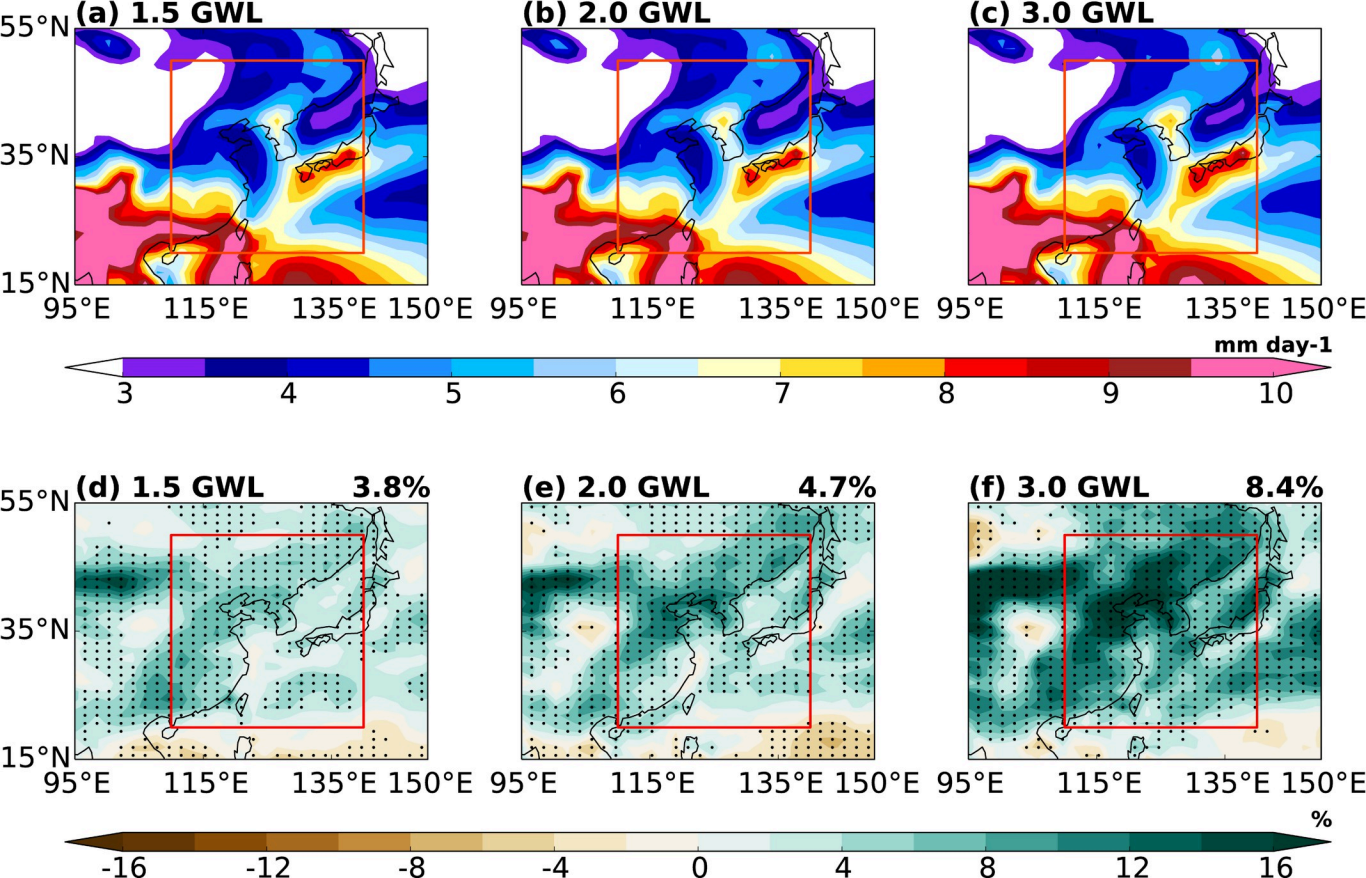
Spatial distributions of future EASM under (a) 1.5°C, (b) 2.0°C, and (c) 3.0°C levels of global warming and (d, e, f) difference of each warming level relative to the PD period. Dotted area indicates significance at 95% confidence level.

Fig 6 shows the spatial distribution of future EASM under 1.5, 2.0, and 3.0°C GWLs. Although the periods of 1.5 and 2°C projected warming are not far from the present period (Section 2.3), a general increase in summer precipitation appears over the EA domain (Fig 6), which is consistent with previous findings [41, 42]. The prominent increase is mainly located in the low latitudes (~25°N) covering southeast China and the western North Pacific and the high latitudes (~40°N) covering northeast China and the Korean Peninsula (Fig 6D–6F). The precipitation changes approximately by 5.7%°C^−1^, 4.0%°C^−1^, and 3.9%°C^−1^ for the three GWLs, respectively. Although these cannot be directly compared with those in previous studies because the latter analyzed the changes EASM at the end of the 21^st^ century [[13] (4.6%°C ^−1^ in CMIP6 only land monsoon), [63] (6.4%°C ^−1^ in CMIP5)], the projected changes in this study are comparable to those of previous studies.

**Fig 7:**
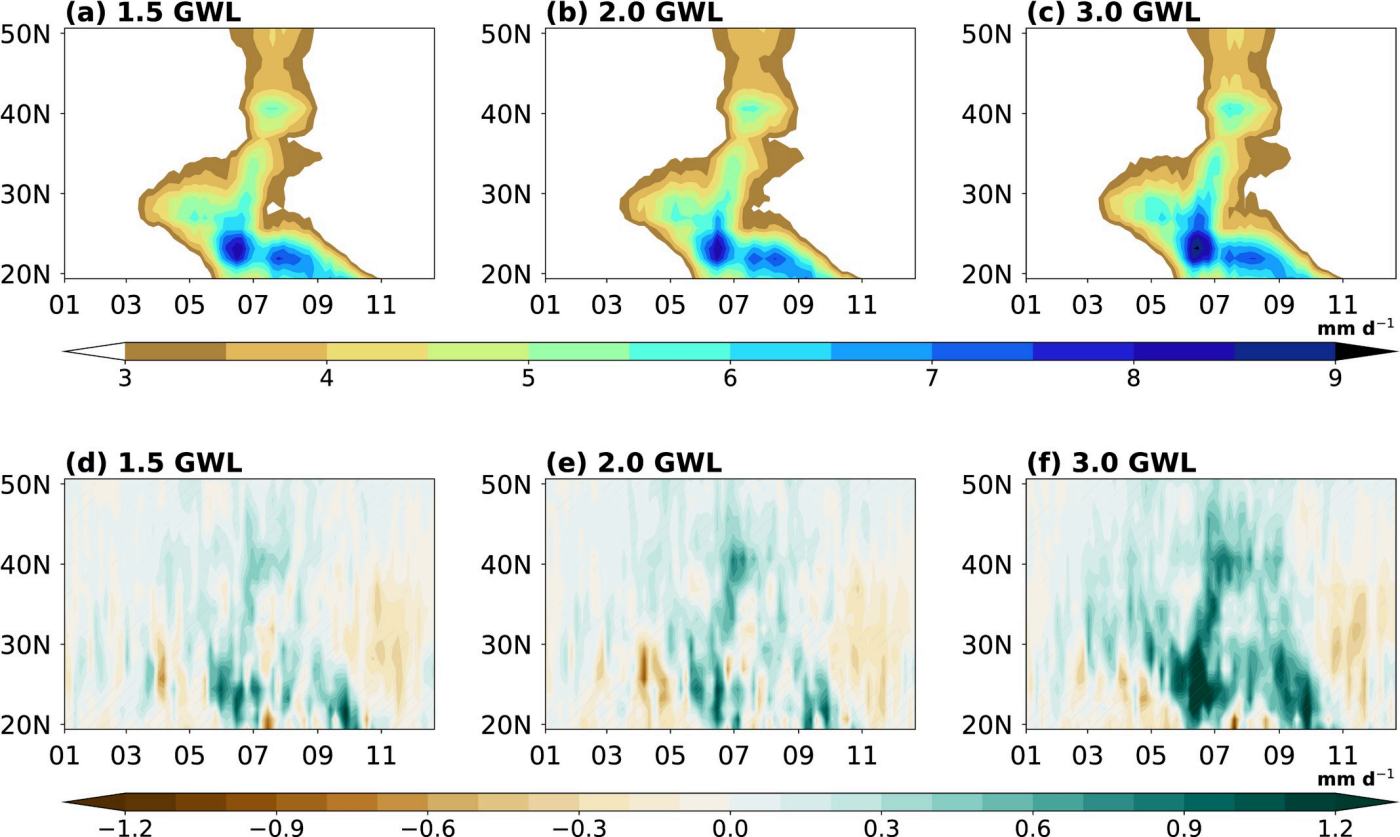
Same as in Fig 2 except for (a) 1.5°C, (b) 2.0°C, and (c) 3.0°C global warming periods and (d, e, f) difference of each warming relative to the PD period. Stippling indicates significance at 95% confidence level.

Fig 7 shows the time–latitude cross section of pentad averaged precipitation anomalies under 1.5, 2.0, and 3.0°C GWLs relative to the PD period. The first appearance of the rainband around 30°N, following enhancement, and abrupt northward movement at the three GWLs are similar to the PD climatology pattern. However, the precipitation amount at each GWL is increased due to rising temperature, and the highest precipitation occurs at 3.0°C GWL. The earlier onset time is approximately one pentad under 1.5°C (33.5 pentad), 2.0°C (33.4 pentad), and 3.0°C (32.9 pentad) warming periods compared with the onset time in the PD climatological conditions (Table 3). Previous studies have calculated the onset time of EASM in the late 21^st^ century; therefore, a direct comparison is difficult. However, previous studies using CMIP3 and CMIP5 have also reported that the onset time is earlier under a warming climate [64–66]. Additionally, the timing of retreat is delayed by approximately 0.2 to 0.7 pentad at 1.5°C (45.4 pentad), 2.0°C (45.4 pentad), and 3.0°C (45.9 pentad) GWLs compared with PD climatological conditions (Table 3). In particular, increasing amounts and expanding precipitation zones occur significantly occurred at 3.0°C warming period (Fig 7C and 7F), and this change is also found in Fig 6C and Table 3. Overall, in the future, EASM has a longer duration (earlier onset time and later withdrawal time; from 0.7 to 1.8 pentad) and larger precipitation (increased amount and expanding zone) with the continuous warming.

**Table 3:** The values for the characteristics of EASM under 1.5, 2.0, and 3.0°C GWLs. The value in parenthesis is change in each warming period compared to the present day (PD) period.

	Onset (pentad)	Withdrawal (pentad)	Duration (pentad)	Amount (mm d^−1^)	Max (mm d^−1^)
1.5°C	33.5 (−0.5)	45.4 (0.2)	11.9 (0.7)	337.8 (25.3)	38.1 (1.6)
2.0°C	33.4 (−0.6)	45.4 (0.2)	12 (0.8)	337.7 (25.2)	37.9 (1.4)
3.0°C	32.9 (−1.1)	45.9 (0.7)	13 (1.8)	372 (59.5)	40.2 (3.7)

**Fig 8:**
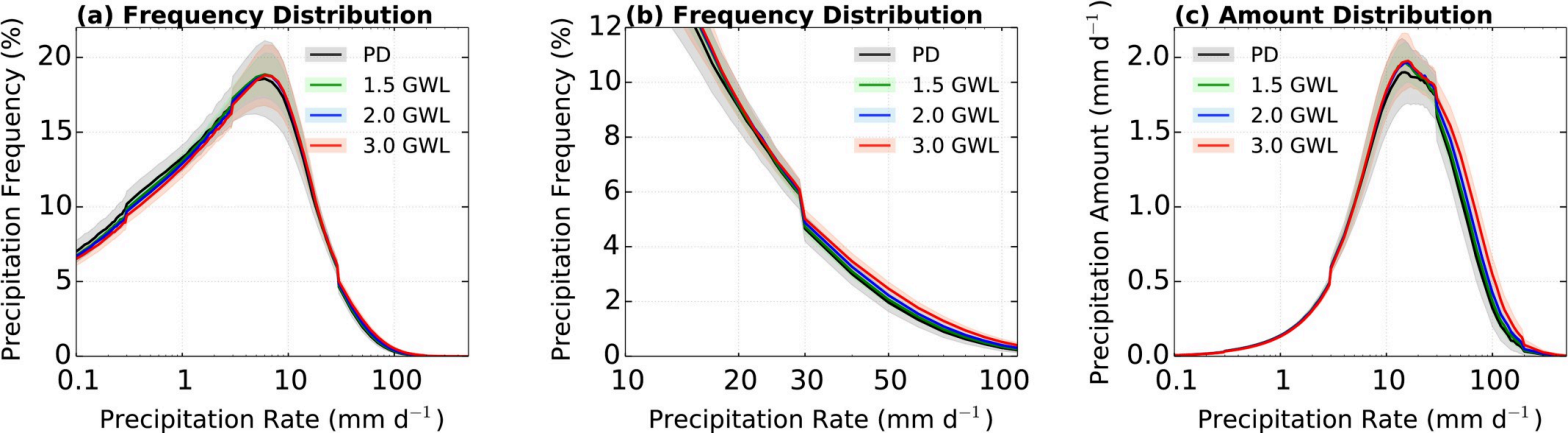
Same as in Fig 4 except for the PD period (black) and 1.5°C (green), 2.0°C (blue), and 3.0°C (red) global warming periods. The shadings are 95% confidence in the CMIP6 ensemble.

The distributions of precipitation frequency and amount are shown in Fig 8 for PD (1995–2014, black) and projected climatology under 1.5, 2.0, and 3.0°C GWLs (green, blue, and red, respectively). Under the three GWLs, the projected distribution of precipitation frequency exhibits a significant decrease in light to weak precipitation rates (below peak; 6 mm d^−1^), and the maximum decrease occurs below 1 mm d^−1^ (Fig 8A). In addition, there is a slight increase above the peak and a significant increase in strong to extreme precipitation rates (Fig 8B; above 29 mm d^−1^; 97th percentile). This analysis is evident in the considerable increase in the strong precipitation frequency due to global warming. Moreover, the projected distribution of amount exhibits a similar signal. Above 6 mm d^−1^, there is an increase in distribution of precipitation amount due to global warming and the maximum increase occurs in strong to extreme precipitation rates (Fig 8C; above 29 mm d^−1^; 97th percentile). Despite the decrease in weak rates and increase in strong rates, both the PD and projected distribution of precipitation amount have similar peaks at approximately 16 mm d^−1^ (92th percentile). Overall, the analysis results indicate that the precipitation changes in the EASM due to global warming mainly occur at precipitation rates above the 97th percentile. In other words, the extreme precipitation (top 3th percentile) in EASM is important component in identifying future changes in EASM.

**Fig 9:**
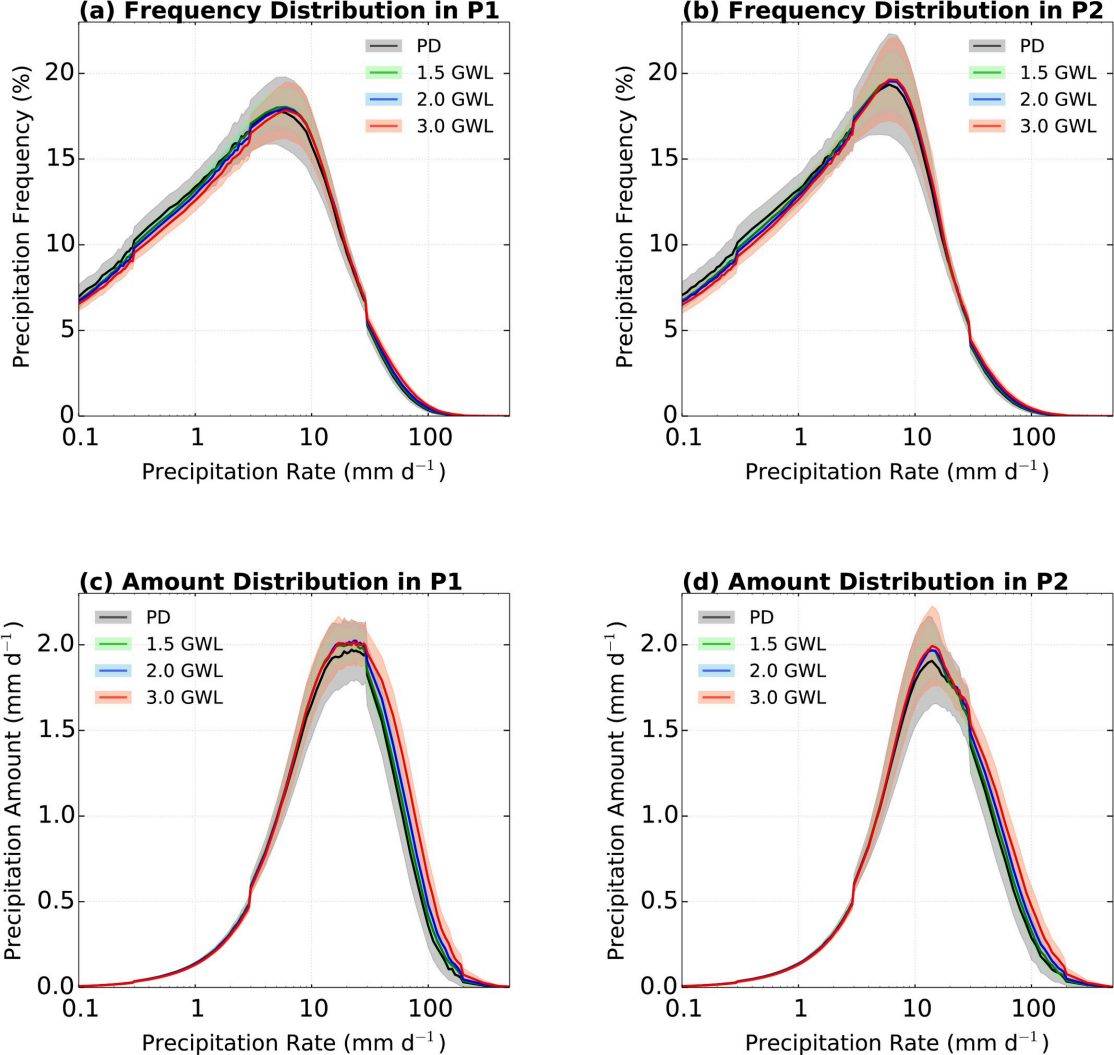
Daily precipitation over the EA domain for distributions of (a, b) precipitation frequency (%) and (c, d) precipitation amount (mm d^−1^). The left and right columns indicate respective distributions in P1 and P2. The black, green, blue, and red lines indicate the PD period, 1.5, 2.0, and 3.0°C global warming periods, respectively. The shadings are 95% confidence in the CMIP6 ensemble.

Fig 9 shows the projected precipitation frequency and amount distributions for P1 and P2. The projected distributions of precipitation frequencies in both periods agree, with a similar peak at approximately 6 mm d^−1^. The tendency of the distribution of precipitation frequency is similar in the entire range of the total period (Fig 8A), while the largest changes occur at the 3.0°C GWL (Fig 9A and 9B). In addition, a higher precipitation amount compared to the PD period is occurred above 6 mm d^−1^ at all GWLs, and these changes mainly correspond to above 97^th^ percentile precipitation rates (Fig 9C and 9D).

## Discussion and conclusion

This study investigates the characteristics of PD and reliable future changes in the EASM at 1.5, 2.0, and 3.0°C GWLs using newly released 30 CMIP6 models with historical and SSP scenarios (SSP1-2.6, SSP2-4.5, SSP3-7.0, and SSP5-8.5). Under the PD climatology in historical simulations, CMIP6 models are able to reproduce precipitation relatively well with a good correlation of 0.91 compared to previous CMIP studies [57 (0.79), 13 (0.88)]. Despite of this improvement, simulated distribution of precipitation frequency at precipitation rates for 1–16 mm d^−1^ (40–92th percentile) is higher than that of observation, and this tendency is mainly occurred in the P2 period. Likewise, precipitation amount distribution at above 16 mm d^−1^ (above 92th percentile) in P1 period lead lower simulated precipitation compared to observation. The precipitation of EASM would increase under global warming based on CMIP6 scenarios with approximately 5.7%°C^−1^, 4.0%°C^−1^, and 3.9%°C^−1^ for the three GWLs, respectively, which is accordance with previous findings. This enhanced precipitation is a result affected by the earlier onset time and delayed retreat time in warmer climate compared to PD climatology. Additionally, the increase of extreme precipitation above 29 mm d^−1^ (97th percentile) is evident that enhanced precipitation of EASM corresponding to GWLs. The changes for distributions of precipitation frequency and amount are occurred in both P1 and P2 periods, while changing amplitudes are the most in the 3.0°C GWL.

Although CMIP6 projections for three GWLs exhibit general increase in EASM precipitation, it is important to note that uncertainty of MME cannot be ignored in this projection. Owing to the complex spatiotemporal characteristics of the EASM system, the effect of common bias of PD on future projections remains unclear. On the other words, we could not report that underestimation bias of PD would lead to underestimated projection. Given the limitation of this projection, four-step strategy adopted in this study may reduce uncertainty of MME and provide more reliable projections. Considering this, the enhanced precipitation caused by an additional 1.0°C warming at 2.0°C GWL are higher than that of the additional 0.5°C warming at 1.5°C GWL (Table 3). This result indicates that continuously rising temperatures can lead to an increase in monsoon precipitation and this tendency would accelerate at higher GWLs. In particular, the distributions of precipitation frequency and amount of strong to heavy precipitation rates (above 29 mm d^−1^; 97th percentile) are mainly increased in warmer climate. This finding provides new insight into water resource management and disaster prevention in summer season of East Asia region. Because, previous studies have projected at the several future periods (e.g. 2041–2060 or 2081–2100) using extreme indices of precipitation and studied general enhancement (not specific season or period). Unexpected extreme precipitation in summer not only lead flood disasters, but also cause linked problems in human society. Our findings are expected to obtain information that can be implemented for sustainable water management programs as a part of the national climate policy.
